# Thromboembolic complications of thyroid storm

**DOI:** 10.1530/EDM-13-0060

**Published:** 2014-02-01

**Authors:** T Min, S Benjamin, L Cozma

**Affiliations:** 1 ST4 Diabetes and EndocrinologyPrincess of Wales HospitalBridgend, CF31 1RQUK; 2 Consultant Diabetes and EndocrinologyPrincess of Wales HospitalBridgend, CF31 1RQUK

## Abstract

Thyroid storm is a rare but potentially life-threatening complication of hyperthyroidism. Early recognition and prompt treatment are essential. Atrial fibrillation can occur in up to 40% of patients with thyroid storm. Studies have shown that hyperthyroidism increases the risk of thromboembolic events. There is no consensus with regard to the initiation of anticoagulation for atrial fibrillation in severe thyrotoxicosis. Anticoagulation is not routinely initiated if the risk is low on a CHADS2 score; however, this should be considered in patients with thyroid storm or severe thyrotoxicosis with impending storm irrespective of the CHADS2 risk, as it appears to increase the risk of thromboembolic episodes. Herein, we describe a case of thyroid storm complicated by massive pulmonary embolism.

**Learning points:**

- Diagnosis of thyroid storm is based on clinical findings. Early recognition and prompt treatment could lead to a favourable outcome.
- Hypercoagulable state is a recognised complication of thyrotoxicosis.
- Atrial fibrillation is strongly associated with hyperthyroidism and thyroid storm.
- Anticoagulation should be considered for patients with severe thyrotoxicosis and atrial fibrillation irrespective of the CHADS2 score.
- Patients with severe thyrotoxicosis and clinical evidence of thrombosis should be immediately anticoagulated until hyperthyroidism is under control.

## Background

Thyroid storm is rare and can be life threatening with a mortality rate of 10–20%; hence, it is important to recognise it early and to initiate appropriate treatment. Atrial fibrillation is a common clinical feature of hyperthyroidism and thyroid storm. However, hyperthyroid status is not taken into account while considering anticoagulation for atrial fibrillation. Studies have demonstrated that hyperthyroidism; especially thyroid storm, is associated with an increased risk of thromboembolic events.

## Case presentation

A 50-year-old man was referred to the medical take with a history of increasing confusion and shortness of breath. He had a background history of schizophrenia, which was well controlled with antipsychotics. He has never smoked and is a teetotaller. No significant past medical history or family history was noted. Within a few hours of assessment, he became agitated and restless.

On examination, he appeared very anxious and had fine tremors in hands, exophthalmos and a diffusely enlarged goitre. He was in atrial fibrillation with heart rate of 165 beats/min, hypotensive with blood pressure of 90/54 mmHg and hypoxic requiring high-flow oxygen to maintain oxygen saturation of 92–94%. He had a raised jugular venous pressure (JVP), mild ankle oedema and equal air entry to both lung fields. A bedside echocardiogram revealed a dilated right ventricle and pressure overload in the right ventricle. A clinical diagnosis of pulmonary embolism was made. As he was in a peri-arrest state, he was thrombolysed as per local hospital protocol. Subsequently, computed tomography pulmonary angiogram (CTPA) confirmed the diagnosis of pulmonary embolism, and he was moved to intensive therapy unit (ITU) for further management.

Thyroid function test showed him to be severely thyrotoxic. On Burch & Wartofsky (1) scoring, he obtained a score of 75: delirium, 20; unexplained jaundice, 20, tachycardia of more than 140 beats/min; 25, atrial fibrillation and ten, suggesting that he was in thyroid storm. A score of 45 or more suggests thyroid storm.

## Investigation

**Figure 1:**
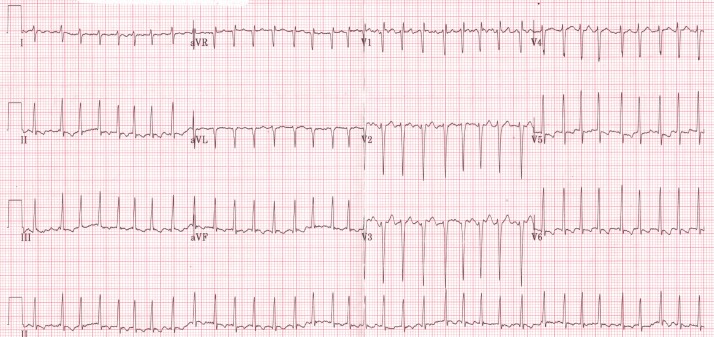
Initial electrocardiogram (ECG) at presentation to the emergency room: heart rate 160 beats/min and atrial fibrillation.

Investigation revealed deranged liver function, normal kidney function and severe thyrotoxicosis with suppressed thyroid-stimulating hormone (TSH) concentration of <0.03 mU/l, free thyroxine (T_4_) concentration of 71.4 pmol/l and free triiodothyronine (T_3_) concentration of 27.4 pmol/l. The concentration of TSH receptor antibody was markedly elevated at 38.2 U/l (<1.0). Atrial fibrillation with a rapid ventricular response (heart rate of 165 beats/min) was noted on ECG (Fig. 1).

**Figure 2:**
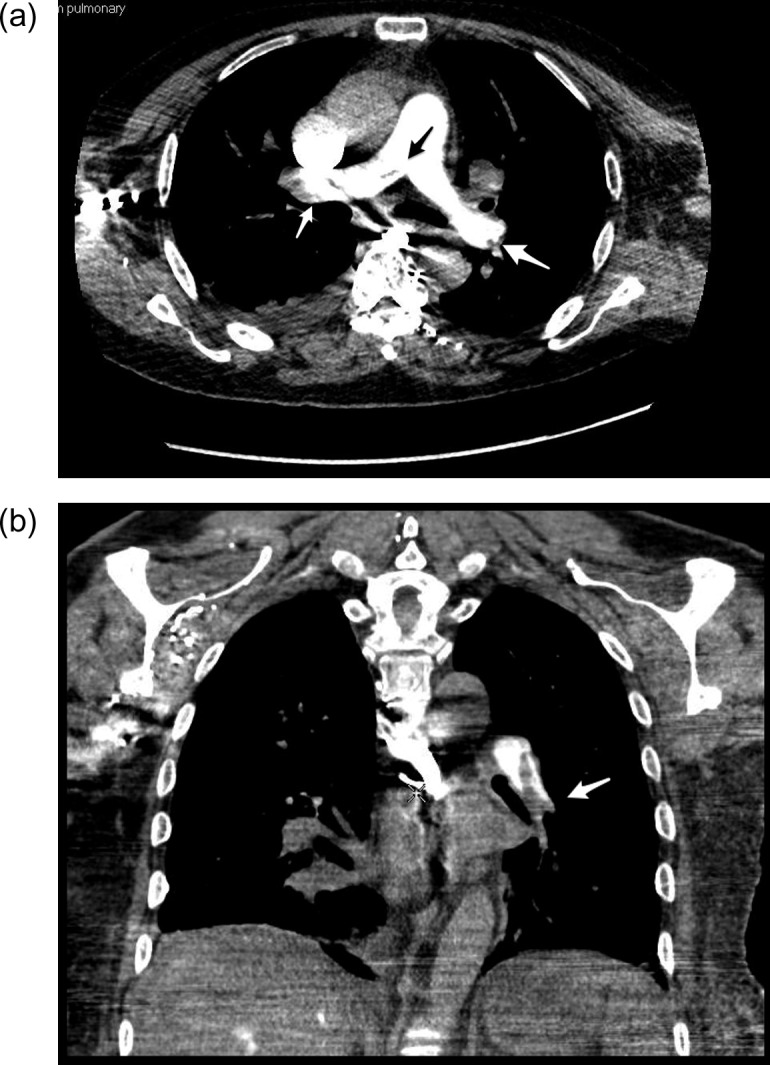
(a) Axial CTPA illustrating a filling defect at the bifurcation of the main pulmonary artery (pointed by black arrow) as well as clots bilaterally (pointed by two white arrows). (b) Coronal view of CTPA showing a large filling defect in the left pulmonary trunk (pointed by white arrow).

Bedside echocardiogram demonstrated globally impaired left ventricle function, dilated right ventricle and pressure overload on the right side of the heart. Subsequent CTPA confirmed multiple bilateral pulmonary emboli (Fig. 2a and b).

## Treatment

He remained in the ITU. Treatment with i.v. steroid and 20 mg OD carbimazole was initiated. Anticoagulation with low-molecular-weight heparin (1.5 mg/kg per day enoxaparin) was continued.

## Outcome and follow-up

The patient made a significant recovery after a week of ITU stay. He was discharged with advice to continue using carbimazole 20 mg OD and enoxaparin 1.5 mg/kg per day s.c.. Repeat thyroid function test done 4 weeks after carbimazole treatment revealed completely suppressed TSH concentration of <0.03 mU/l with normal free T_4_ concentration of 16.4 pmol/l and normal free T_3_ concentration of 4.8 pmol/l.

## Discussion

Thyroid storm is rare and can be life threatening with a mortality rate of 10–20%; hence, it is important to recognise it early and initiate appropriate treatment. The cause for thyrotoxicosis in thyroid storm is likely due to Graves' disease; however, it occurs with other forms of thyrotoxicosis.

The diagnosis of thyroid storm is based on clinical findings. Burch & Wartofsky (1) developed a scoring system based on clinical criteria in 1993. A score between 25 and 44 is suggestive of impending thyroid storm and a score >44 is suggestive of thyroid storm. More recently, Akamizu *et al*. (2) have formulated diagnostic criteria based on the review of clinical manifestations, namely CNS, cardiovascular, gastrointestinal and hepatic manifestations and multi-organ failure. The prerequisite for the diagnosis of thyroid storm is a definite biochemical evidence of thyrotoxicosis. In the study carried out by Akamizu *et al*., a 10% mortality rate was reported for thyroid storm and the presence of multi-organ failure was an independent prognostic factor for death. A higher incidence of irreversible neurological defects including psychosis was observed in this cohort.

Precipitating factors for thyroid storm are poor compliance with medications, delayed treatment, infection, radioiodine therapy, trauma, surgical procedures and cardiovascular events. Individuals at a risk of developing thyroid storm are elderly, individuals with mental health issues, pregnant women, individuals with Addison's disease and individuals with alcohol abuse.

The pathophysiology of thyroid storm is unclear; the hypothesis suggested is an increase in free T_3_ concentrations and increase in β-adrenergic receptor activation. There is usually a precipitating event that sets off the thyroid storm. There is no clear cut-off level for free T_4_ or free T_3_ to predict severe thyrotoxicosis. What is important is to recognise the severity of thyrotoxicosis and treat the patient appropriately.

Previous studies have reported that hyperthyroidism is associated with a hypercoagulable state. There are case reports of sinus, cerebral thrombosis and deep vein thrombosis (3) in patients with thyrotoxicosis. The hypercoagulable state may be multifactorial and be linked to an increase in the activity of factor VIII, Von Willebrand factor and tissue plasminogen activator inhibitor-1 (4). A 5-year follow-up study carried out by Lin *et al*. (5) to assess the risk of pulmonary embolism in hyperthyroid patients found a 2.3 times greater increase in the risk of pulmonary embolism after removing the confounding factors using Cox model hazard regression.

Atrial fibrillation is a clinical feature of hyperthyroidism with an estimated prevalence rate of 10–20% in patients with thyrotoxicosis (2) (6) (7); however, in thyroid storm, a 30–40% occurrence rate of atrial fibrillation has been observed and 50% of patients who died due to thyroid storm have been reported to be in atrial fibrillation (2). About 10–40% of hyperthyroid patients with atrial fibrillation have been found to have arterial thrombosis (8). There is no consensus with regard to the initiation of anticoagulation for atrial fibrillation in severe thyrotoxicosis. Anticoagulation is not routinely initiated if the risk is low on the CHADS2 score; however, this should be considered in patients with thyroid storm or severe thyrotoxicosis with impending storm irrespective of the CHADS2 risk, as it appears to increase the risk of thromboembolic episodes.

## Patient consent

Written informed consent was obtained from the patient for publication of the case report and accompanying images.

## Author contribution statement

T Min was responsible for case note review and literature search and wrote the case report; S Benjamin reviewed the patient in the ward and while visiting the outpatient clinics and L Cozma was responsible for discussion and literature review.
